# Editorial for “Regulatory RNAs in the nervous system”

**DOI:** 10.3389/fncel.2015.00038

**Published:** 2015-02-10

**Authors:** Alessandro Cellerino, Laure Bally-Cuif, Tommaso Pizzorusso

**Affiliations:** ^1^Scuola Normale SuperiorePisa, Italy; ^2^Biology of Aging, Fritz Lipmann Institute for Age Research-Leibniz InstituteJena, Germany; ^3^Institute of Neurobiology A. Fessard, CNRS UPR3294Gif-sur-Yvette, France; ^4^Department of Neuroscience, Psychology, Drug Research and Child Health Neurofarba, University of FlorenceFlorence, Italy; ^5^Pisa Unit, Institute of Neuroscience, National Research CouncilPisa, Italy

**Keywords:** microRNA, non-coding RNA, neuronal development, neuronal plasticity, aging

Until about a decade ago, the non-coding part of the genome was considered without function. The development of high-throughput RNA sequencing techniques (next-generation sequencing) revealed the existence of many transcripts that do not code for proteins in addition to the RNA components needed for mRNA translation: rRNAs and tRNAs. The aim of this issue was to put together reports on the role of non-coding RNAs in the nervous system, an emerging field not covered so far in a systematic manner.

Non-coding transcripts can be divided into three broad classes: (i) short RNAs (sRNAs), (ii) RNAs transcribed from the opposite strand of a protein-coding locus that contain sequences antisense with respect to the protein-coding transcript, (OS-RNAs) and (iii) long intergenic non-coding RNAs (lincRNAs). Many of these non-coding RNAs (nc-RNAs) can regulate the transcription or the translation of protein-coding genes. Almost on weekly basis, new findings reveal the regulatory role that nc-RNAs exert in many biological processes. Overall, these studies are making increasingly clear that, both in model organisms and in humans, complexity is not a function of the number of protein-coding genes, but results from the possibility of using combinations of genetic programs and controlling their spatial and temporal regulation during development, senescence and in disease by regulatory RNAs. This has generated a novel picture of gene regulatory networks where regulatory nc-RNAs represent novel layers of regulation. Publications reporting novel non-coding RNAs found using sequencing appears almost monthly, therefore dedicated bioinformatics techniques to analyze the result of this analysis are under development (Guffanti et al., 2014).

Particularly well-characterized is the role of microRNAs (miRNAs) in the post-transcriptional regulation of gene expression. MicroRNAs are short(~21 nt) nc-RNAs that arise from processing of a long primary transcript via a complex and well-described biosynthetic process. MicroRNAs bind to mRNAs (usually in the 3′untranslated region) and regulate gene expression by repressing mRNA translation and/or inducing degradation of the target mRNA. Up to now, several thousands of miRNAs have been predicted and identified in animals, plants and viruses (www.mirbase.org) and some microRNAs are highly conserved, facilitating the analysis of microRNA in non-model species. A feature of miRNAs is their combinatorial regulation: a given miRNA can target a multitude of different mRNAs and a given target might similarly be targeted by multiple miRNAs; for this reason, they frequently represent the central nodes of several regulatory networks and may act as rheostat to provide stability and fine-tuning to gene expression networks (Osella et al., 2011; Siciliano et al., 2013). MicroRNAs are also relatively easy to study experimentally and novel methods to study their function are continually coming out (Chaudhuri et al., 2013; Knauss et al., 2013). They can be transfected in cells, microinjected in embryos or delivered *in vivo* to neurons and their function can be blocked, *in vitro* and *in vivo*, by modified antisense oligonucleotides (antagomiRs). For all these reasons, the majority of contributions to this e-book relate to miRNAs. In the nervous system, miRNAs have been involved in the regulation of cellular pathways controlling fundamental functions during development (Benchoua and Peschanski, 2013; Coolen et al., 2013; Cremisi, 2013; Hong et al., 2013; Iyengar et al., 2014; Iyer et al., 2014; Terzibasi Tozzini et al., 2014), synaptic plasticity (Tognini and Pizzorusso, 2012; Chiu et al., 2014), and in neurodegenerative disease. Intriguingly, miRNAs show a double-sided relationship with neuronal activity: electrical activity (Eacker et al., 2013; Pai et al., 2014) regulates miRNAs at the level of transcription, biogenesis, stability and specific targeting to dendrites and also axons and presynaptic terminals (Kaplan et al., 2013) on one side, but miRNAs are also able to regulate membrane conductances altering neuronal biophysical properties (Gavazzo et al., 2013). Synaptic localization is particularly relevant in the context of local translational control (Heise et al., 2014), thereby providing a molecular substrate for synaptic plasticity. Deregulation of expression of miRNAs is proposed not only as potential disease biomarker (Sheinerman and Umansky, 2013; Maffioletti et al., 2014), but it has been implicated directly in the pathogenesis of complex neurological and neuropsychiatric disease (Dogini et al., 2013; Goodall et al., 2013; Maciotta et al., 2013; Serafini et al., 2013; Barbato et al., 2014; Della Ragione et al., 2014; Elramah et al., 2014; Fragkouli and Doxakis, 2014; Kye and Goncalves Ido, 2014; Nieto-Diaz et al., 2014). This so-called RNA revolution also lead to the exploitation of RNA interference and the development of related tools as potential treatment of a vast array of CNS disease that could benefit from regulation of disease-associated genes.

A second class of small RNAs are the piwi-interacting RNAs (piRNAs). These are slightly larger than miRNAs (24–32 nt) originate from intergenicrepetive sequences that are transcribed as a long RNA and processed and play an important role in gametogenesis and transposon silencing. PiRNAs are expressed at low level (if at all) in somatic tissues and their role in the nervous system is still ill-characterized.

Long non-coding RNAs are a heterogeneous population and are much less studied (see Ernst and Morton, 2013). They can be associated to chromatin and either interfere with transcription of the target gene(s) or induce epigenetic modifications. Long ncRNAs can indeed interact with chromatin remodellers such as Polycomb and target these to specific genomic regions. Opposite-strand RNAs can hybridize with their protein-coding complementary transcript and modulate splicing or induce RNA degradation. Finally, long ncRNAs derived from pseudogenes can act as competitive inhibitors for miRNAs thereby increasing the expression of their protein-coding paralog. Examples of these mechanisms relate to transcription of repetitive elements (Pascarella et al., 2014) or fine tuning of developmental patterning and positional information in the central nervous system mediated by regulation of the spatial pattern of expression of Hox genes in Drosophila (Gummalla et al., 2014).

## Conflict of interest statement

The authors declare that the research was conducted in the absence of any commercial or financial relationships that could be construed as a potential conflict of interest.
